# The courses of objective physical activity and the association with sleepiness during a 2-week-on/2-week-off offshore shift rotation: an observational repeated-measures study

**DOI:** 10.1186/s12889-021-10756-2

**Published:** 2021-04-17

**Authors:** P. Ots, V. Riethmeister, J. Almansa, U. Bültmann, S. Brouwer

**Affiliations:** Department of Health Sciences, Community and Occupational Medicine, University of Groningen, University Medical Center Groningen, Hanzeplein 1, 9700 RB Groningen, The Netherlands

**Keywords:** Physical activity, Sleepiness, Offshore

## Abstract

**Background:**

Offshore workers are assumed to have poor health behaviours, but no studies have yet examined physical activity (PA) during a full offshore shift rotation period, including both work and at home periods. Furthermore, the relationship of PA with sleepiness, a prevalent safety hazard offshore, is not known. This study aimed to examine (1) the courses of objectively measured PA in offshore workers during pre-, offshore and post-offshore periods, and (2) the association between PA and self-reported sleepiness.

**Methods:**

An observational repeated measures study was conducted among 36 offshore workers during a full 2-week on/2-week off offshore shift rotation. Objective PA was assessed using Daytime Activity Averages (DAA) from actigraph recordings. Sleepiness was assessed using next-morning Karolinska Sleepiness Scale (KSS) scores. The courses of PA over time were analysed with Linear Mixed Models (LMM). Parallel LMM were used to assess the longitudinal relationship between PA and sleepiness, both on a between-person and within-person level.

**Results:**

The courses of PA were not significantly different between the pre-, offshore, and post-offshore periods. In addition, between-person trends of PA and sleepiness were not associated (*p* ranges between 0.08─0.99) and PA did not affect next-morning sleepiness on a within-person level (*p* = 0.15).

**Conclusions:**

PA levels during the offshore working period were not different from PA levels at home. Furthermore, PA was not associated with next-morning sleepiness. Further research should focus on different levels of PA including its intensity level.

## Background

Physical activity (PA) is an important health behaviour which is often defined as ‘any bodily movement produced by skeletal muscles that results in energy expenditure’ [1, 2]. PA has been shown to be essential in reducing the risk of a number of chronic health conditions among which musculoskeletal complaints [3]. Furthermore, it has the potential to reduce the likelihood of developing obesity [4], can increase workers’ productivity [5], and may reduce work-related fatigue [6]. Knowledge on PA in constrained environments, such as the offshore environment, is limited [7].

Offshore workers operate on remote oil and gas locations with unique occupational hazards, such as extreme weather conditions and the operation of heavy machinery. The main aim of the offhore work environments is oil and gas production. The platforms are accesible by helicopters. Specific physical characteristics of the platforms (e.g. noise and motion) and social characteristics of the job (e.g., being away from family) are unique within this working environment. In the Dutch offshore environment, most offshore workers work in 2-weeks on/2-weeks off shift rotations. Typically, shifts last for 12 h, from 7 AM to 7 PM, for 14 consecutive days offshore followed by 14 days of leave at home. Due to physically demanding work conditions, having a good health is required to work offshore. A sedentary lifestyle, including unhealthy eating behaviors, is however common [8, 9]. Although research has indicated that offshore workers consider exercise as a health-related strategy to cope with job demands [10] or manage their fatigue [11], it has also been shown that PA levels on offshore platforms are to a large extent sedentary [12]. Offshore inactivity generally results from inadequate gym facilities, low interest in exercising, or fatigue [13]. Furthermore, it has been shown that workers engaged in PA during their leave periods to promote recovery [14]. All studies were qualitative or used self-reported PA data. As PA has not been objectively investigated across an offshore shift rotation, it remains largely unknown whether offshore workers’ PA levels change over time, i.e. during and across the pre-offshore, offshore, and post-offshore periods. Examining these different periods would increase knowledge on differences between PA during the offshore working period and leave periods at home. Furthermore, it could possibly serve as a basis for the development of physical activity promotion programs offshore.

In the offshore environment, fatigue has been identified as one of the main safety hazards [15]. Sleepiness is an important predictor of injuries and insomnia symptoms are significant predictors of work accidents [16]. In an earlier study, we demonstrated that especially self-reported fatigue increased during the offshore period [17]. In addition, workers slept less whilst offshore, rated their sleep quality lower, and felt less refreshed after awakening [18]. A relatively high work load, shift work, and long work hours are characteristics of the offshore work environment associated with higher fatigue levels [19, 20]. Previous research has also shown that PA is longitudinally associated with sleepiness, yet findings are inconsistent depending on how PA and sleepiness are defined [6, 21–23]. In some cases, physical activity is defined as being active at work in physically strenuous jobs, which has been associated with higher sleepiness levels. For instance, Arias et al. showed that objective actigraph recordings of occupational PA were associated with higher levels of work-related fatigue among construction workers after 1 week [21]. In contrast, other studies have focussed on leisure-time activities or exercise when investigating PA [22, 23]. A Dutch diary study among a diverse group of workers (e.g. IT-workers and workers from a petroleum company) showed that the more time workers spent on PA during leisure time, the lower their experienced fatigue [22]. Furthermore, workplace health promotion programs stimulating PA have been found to decrease sleepiness or the risk of developing fatigue [24, 25]. This could be explained the amount of slow wave sleep, which is assumed to be higher among those that regularly engage in PA compared to those who do not regularly exercise [24]. Moreover, the onset of REM sleep might be delayed and total sleep time might be prolonged, which all may contribute to lower levels of sleepiness or fatigue [25]. The association between PA and fatigue has not yet been examined among offshore workers. If PA plays a role in the course of fatigue over time, the effectiveness of PA interventions could be investigated in future studies, and could potentially be implemented in future fatigue risk management programs. 

The current study aimed to examine (1) the courses of PA during the pre-, offshore and post-offshore period and (2) the association between PA and sleepiness across the full offshore shift rotation period.

## Methods

### Design and procedure

This study used data from the ‘Offshore Sleep and Fatigue Study’, a prospective cohort study with repeated measures, conducted from February to June 2015 [17]. Recruitment of offshore workers took place through invitation emails and study promotion displayed on four selected offshore platforms in the Netherlands and the United Kingdom. Platform location is not elucidated further for the purpose of confidentiality. Data was collected 1 week before the offshore period (pre-offshore, days 1–7), during the two-week offshore period (offshore, days 8–21), and 1 week after the offshore period (post-offshore, days 22–28). Baseline data was obtained by an online questionnaire at the first day of the study. After waking up on study day 1, participants were asked to wear the actigraph (MotionWatch 8), a movement tracking device, and to wear the device throughout the entire study period. Sleepiness was assessed twice a day using an online questionnaire. As daily measurements were administered for 28 days, this resulted in 1008 total measures for both PA and sleepiness. Additional information about the study can be found elsewhere [17, 18].

### Participants

**Fig. 1:**
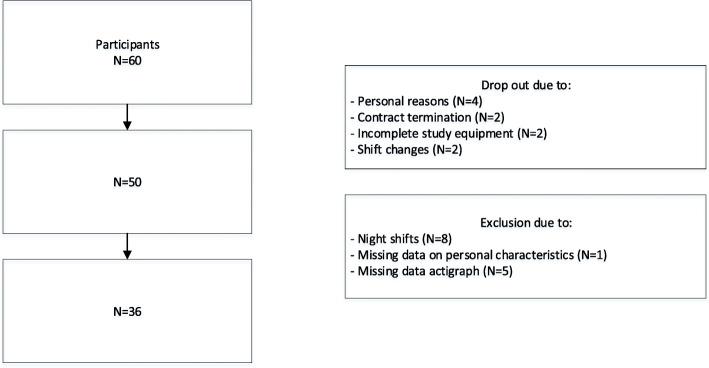
Flow chart of participants selection

Offshore workers from Dutch and British offshore platforms participated in the study. The offshore workers operated in a 2-week on/2-week off offshore shift rotation, working 14 consecutive days on the offshore platform followed by 14 days of leave. The offshore work schedule consisted of 12-h shifts starting at 7:00 AM and ending at 7:00 PM. In total, 60 workers volunteered from the target population to participate in the study. The company allowed workers to participate in the study during working hours. Of the 60 participants that initially agreed to participate in the study, ten participants could not start due to personal reasons, contract termination, incomplete study equipment or shift changes. Of the 50 remaining participants, we included 36 day-shift workers. We excluded 14 participants because 1) they worked night shifts (*N* = 8) and 2) they had missing data on some of the key variables (*N* = 6). On the physical activity measures, assessed with the actigraph, five participants did not have any scores at all. Additionally, one participant did not fill in the baseline questionnaire (Fig. 1). As daily measurements were administered for 28 days, this resulted in 1008 total measures for both PA and sleepiness. Ethical approval was obtained from the Medical Ethics Committee of the University Medical Center Groningen, The Netherlands (reference number: M14.165646).

### Measures

#### Physical activity

Objective daytime PA was assessed using wrist worn actigraph recordings (MotionWatch 8, CamNtech) [26]. The MotionWatch (MW) 8 is a lightweight compact device that monitors movement throughout the day and night. Before the start of the study, offshore workers received information on the use of the device and were asked to wear the device on their non-dominant wrist throughout the full offshore shift rotation of 28 days, including the offshore work period and the leave period at home. Daytime activity was recorded from 6 AM to 11 PM using 60s epochs. Daytime activity average (DAA) scores were calculated based on MW 8 counts per minute.

#### Sleepiness

Sleepiness was assessed with morning ratings of the Karolinska Sleepiness Scale (KSS) during the full 2-week on/2-week off offshore shift rotation [27]. The KSS comprises one self-reported item, assessed on a 9-point Likert scale, ranging from [1] ‘very alert’ to [9] ‘very sleepy, fighting sleep and effort to stay awake’. Higher scores indicate higher levels of sleepiness. For the analyses and interpretation of the graphical representation, scores were multiplied by 10.

#### Socio-demographics, health and health behaviours

Data on age, nationality, and platform location were taken from the baseline questionnaire. Job function was based on a description of participants’ work activities and categorized into operations, maintenance, supervision, catering, safety, and other. Self-reported health was assessed using the one-item short-form survey (SF-1) of the original SF-12 questionnaire: ‘In general, would you say that your health is… ‘excellent’, ‘very good’, ‘good’, ‘fair’, or ‘poor’ [28]. Data were dichotomized into good health (excellent/very god/good`) and fair/poor health. Body Mass Index (BMI) was calculated using the formula: weight (kg) / [height (m)^2^, and was categorized according to WHO guidelines into: ‘underweight’ (BMI < 18.5), ‘normal weight’ (BMI ≥18.5 to ≤24.9), ‘overweight’ (BMI ≥25 to ≤29.9) and ‘obesity’ (BMI ≥30). Smoking was examined by asking ‘Do you smoke?’ (no/yes, around _ packs per week’). Alcohol consumption was assessed by asking ‘Do you consume alcoholic beverages when at home?’ (no/yes, I do drink alcohol. On average, _ glasses per week’. Sleep quality was assessed using night-time activity average (NAA) scores retrieved from objective actigraph recordings with the MW 8 [26].

### Statistical analysis

First, descriptive statistics (e.g., means and standard deviations) were presented for the sample characteristics. Second, the PA (DAA) courses during the full offshore shift rotation were examined using linear mixed models (LMM) with random intercept and a random linear slope for day. The PA course (slope and intercept) estimations were allowed to vary within the pre-offshore, offshore, and post-offshore periods, and were shown for each period. If no period-specific differences were observed, the model was re-estimated with unique parameters over all days. Third, a parallel LMM analysis was performed to examine the association between PA and next-morning sleepiness (KSS). Parallel LMM is a multivariate extension of LMM, in which both variables are modelled over time, allowing their intercepts and slopes to covary [29]. At the between-person level, the model assesses to what extent the trends over time (random intercept and slope) of PA correlate with the trends in sleepiness across all individuals. At the within-person level, the model assesses if variations in PA beyond each individual’s expected value are associated with variations beyond the expected next-morning sleepiness value for a specific day. The within-person associations between PA and sleepiness were adjusted for sleep quality, which was added to the model as a time-varying covariate. In addition, all models were adjusted by age and platform location.

For all analyses, data was assumed to be missing at random, using maximum likelihood estimation methods and robust standard errors. For all LMM’s, unstructured random effects covariance matrices were used. As the first and last day (day 1 and day 28) of the rotation did not provide stable sleepiness data because of logistical constraints, sensitivity analyses were conducted excluding these days. Analyses were performed using IBM SPSS Statistics version 25 [30] and Mplus version 8 [31]. Findings were considered statistically significant when *p* < 0.05.

## Results

### Sample characteristics

Baseline characteristics of 36 offshore workers were available for data analyses (Fig. 1; Table 1). The mean age of the participants was 44.3 years (*SD* = 11.1). Participants were Dutch (72.2%), British (22.2%) or Australian (5.6%) and most participants had a job function related to maintenance (41.7%) or operations (27.8%). The majority of the participants rated their general health to be good (97.2%). A total of 47.2% of the participants was overweight and 13.9% was obese. In total, 25% of offshore workers smoked and weekly alcohol consumption during pre- and post-offshore periods ranged from 0 to 50 consumptions (*M* = 8.0, *SD* = 9.3). Sample characteristics did not differ between offshore workers who completed the study (*N* = 50) and those included the final sample (*N* = 36). Missing values were observed for DAA (10.3%) and for KSS (11.6%).

**Table 1:** Sample characteristics of offshore workers (*N* = 36)

Age (years) (*M, SD*)	44.3 (11.1)
Nationality (%)	
British	8 (22.2)
Australian	2 (5.6)
Dutch	26 (72.2)
Job function (%)	
Operations	10 (27.8)
Maintenance	15 (41.7)
Catering	1 (2.8)
Supervision	6 (16.7)
Safety	2 (5.5)
Other	2 (5.5)
Perceived general health (SF-1) (%)	
Excellent, very good, good	35 (97.2)
Fair, poor	1 (2.8)
BMI (%)	
Normal weight	14 (38.9)
Overweight	17 (47.2)
Obese	5 (13.9)
Smoking	
Yes (%)	9 (25.0)
Packs/week (*M, SD*)	1.2 (2.1)
Alcohol consumption	
Yes (%)	32 (88.9)
Glasses/week (*M, SD*)	8.0 (9.3)

### Courses of physical activity during the pre-, offshore and post-offshore period

**Fig. 2:**
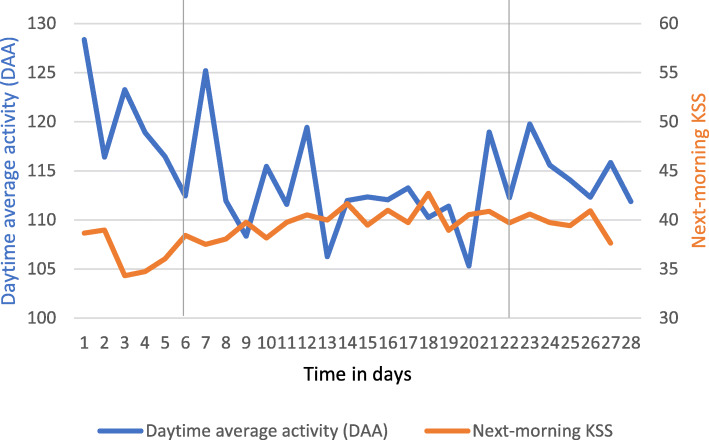
Courses of PA, measured by daytime average activity (DAA) and sleepiness, measured by KSS, across the full offshore shift rotation (*N* = 36). The grey lines differentiate between the pre-offshore (day 1–7), offshore (day 8–21), and post-offshore period (day 22–28). Please note the truncation of the Y-axis; KSS scores multiplied by 10; and the standard deviation not being shown

The courses of PA during the pre-, offshore and post-offshore period did not significantly change over time (Fig. 2; Table 2). The intercept of PA was lowest during the post-offshore period (*b* = 125.7) and a bit higher and similar between the pre-offshore and offshore periods (*b* = 137.8 and *b* = 143.4 respectively). PA decreased during both the pre- and offshore period (*b* = − 0.8 and *b* = − 0.6) and was stable during the post-offshore period (*b* = 0.0), but there was no significant difference between the period-specific slopes. The highest mean PA score was observed at day 1 (*M* = 128.34, *SD* = 64.19) and the lowest score was found at day 20 (*M* = 105.30, *SD* = 32.50). As no significant differences between period-specific intercepts and slopes were found, the overall course across the full offshore shift rotation period was estimated. The overall average course of PA did not significantly change over the 28 days (*b* = − 0.27; *p* = .19).

**Table 2:** Parameter estimates (intercepts and slopes) of daytime average activity (DAA) scores depicting the PA course within the pre-offshore, offshore, and post-offshore period, adjusted for age and platform location (*N* = 36)

	Estimate (SE)	*p*-value
Fixed effects		
Intercept	137.8 (34.4)	0.00
Pre-offshore	ref	
Offshore	5.6 (25.2)	0.82
Post-offshore	−12.1 (11.3)	0.29
Slope (Time in days)	−0.8 (1.6)	0.61
Pre-offshore	ref	
Offshore	0.2 (1.8)	0.91
Post-offshore	0.8 (1.6)	0.60
Random effects		
Intercept variance	723.8 (174.0)	
Slope variance	0.7 (0.5)	
Intercept–slope covariance	−8.3 (9.0)	0.30
Residual variance	975.5 (126.5)	

### The association between PA and next-morning sleepiness

At the between-person level, PA and sleepiness trends were not associated; estimated intercepts and slopes did not covary (*p* ranges from 0.08─0.99) (Fig. 2; Table 3). At the within-person level, PA and sleepiness were not associated (*p* = 0.15). The lowest morning sleepiness score was found at day 4 (*M* = 34.3, *SD* = 14.1) and the highest morning sleepiness score was found at day 19 (*M* = 42.7, *SD* = 17.6).

**Table 3:** Parameter estimates of the parallel longitudinal model of daytime average activity (DAA) and association with next morning sleepiness (KSS) trends, adjusted for age and platform location (*N* = 36)

	Estimate (SE)	*p*-value
**Fixed effects**		
DAA		
Intercept	16.9 (4.7)	0.00
Slope	−0.18 (0.17)	0.32
KSS		
Intercept	3.66 (0.22)	0.00
Slope	0.01 (0.01)	0.14
DAA	0.001 (0.001)	0.15
NAA	−0.002 (0.003)	0.43
**Random effects**		
DAA		
Intercept DAA variance	598.9 (134.1)	
Slope DAA variance	0.27 (0.26)	
Intercept–slope DAA covariance	−0.38 (5.2)	0.94
KSS		
Intercept KSS variance	1.51 (0.43)	
Slope KSS variance	0.001 (0.000)	
Intercept–slope KSS covariance	−0.01 (0.01)	0.31
DAA-KSS		
Covariance intercept DAA–slope KSS	−0.10 (0.18)	0.56
Covariance intercept KSS–slope DAA	0.40 (0.23)	0.08
Covariance intercept DAA–intercept KSS	0.11 (7.2)	0.99
Covariance slope DAA–slope KSS	−0.001 (0.007)	0.89
Residual KSS variance	1.1 (0.15)	
Residual DAA variance	932.3 (125.6)	

### Sensitivity analyses

After excluding day 1 and day 28 from the analyses, the PA course did not vary across the 26 days. The highest PA score was found at day 7 (*M* = 125.19, *SD* = 46.12). No differences between PA courses across periods were found and the association between PA and next-morning sleepiness remained non-significant.

## Discussion

The current study provided insight into the courses of physical activity (PA) among offshore workers during the pre-, offshore, and post-offshore period by using a repeated measures design and an objective tool to examine PA. Results showed that the courses of PA were not significantly different across the three period, i.e. activity levels of offshore workers are comparable at the offshore platform and at home. In addition, daytime PA was not associated with next-morning sleepiness during the offshore shift rotation. Overall, our findings showed robustness and were not affected by the in- or exclusion of the first and last day of the investigation period.

The current study is unique in its use of an objective measure to assess PA during a full offshore shift rotation period. A previous study among offshore workers indicated that self-reported activity levels of day-shift workers slightly decreased during the post-offshore period [32]. Additionally, Mearns et al. showed that offshore workers reported to be more physically active at home than at the platform, with differences ranging from 69% being active at home compared to 50% being active while offshore [12]. In addition, a study among offshore fleet seafarers showed less activity offshore than at home [33]. Although in the current study the intercept of PA during the post-offshore periods was slightly lower than during the pre-offshore and offshore periods, we did not find significant differences between the offshore work period and the pre- and post-offshore periods at home. This is not in line with earlier studies indicating that being physically active can be more difficult offshore than at home. While previous studies assessed PA levels using subjective self-reported measures [12, 14, 33], we used objective actigraph recordings, which may be an explanation. Previous research has indicated that individuals are likely to either over- or underestimate their PA levels, and especially leisure-time PA levels are found to more often overreported [34]. Thus, although workers may report to be more active at home, this may not necessarily be the case.

No association between PA and self-reported sleepiness was found. Previous studies linking PA actigraph recordings with sleepiness reported mixed findings, e.g. one study suggested that PA may reduce fatigue [35], while another one did not find any effects [36]. In the current study, the lack of an association might be explained by the relatively stable course of PA over time. More within-day variation in PA, i.e. larger differences on day-level, may be associated with sleepiness. In addition, there may be other offshore environment characteristics that affect sleepiness, such as long work hours and 14-day shifts in combination with unpleasant sleeping conditions and noise. In addition, future studies may consider examining the prevalence of obstructive sleep apnoea (OSA) in the offshore population. OSA is often a missed cause of sleepiness and linked with obesity and hypertension, which are common among offshore workers [37, 38]. Furthermore, OSA has been shown to impair work functioning [39]. Given that sleepiness is an important workplace hazard offshore, further research into predictors of sleepiness offshore is needed. Potential predictors to be examined could, for instance, include psychological resources depletion, the change of activities, motivation or perceived stress [40].

### Strengths and limitations

A strength of this study is the use of a repeated measures design to examine the course of PA during the offshore period and the pre- and post-offshore period at home. In addition, objective PA was used in this study, which is not influenced by recall bias or socially desirable responses. Moreover, using continuous PA scores of the objective actigraph recordings to assess PA by Daytime Average Activity (DAA) scores is more accurate, as cut-off values of PA (e.g. being moderately active or vigorously active) can lead to under- or over-interpretation of energy expenditure [41]. Furthermore, using parallel LLM analyses to assess the association between PA and sleepiness allowed us to separate the between and within effects, i.e. the day-levels were not confounded by person-level characteristics.

Some study limitations have to be reported. The sample size was rather small and 24 of participants could not be included in the analyses due to early drop-out, changes in work schedules or missing data on key variables. However, the data was collected over the full offshore shift rotation with repeated measures for 36 workers for 28 days, which enabled the use of within-person analyses with 1008 measurements. Next, our sample was restricted to day-shift workers as we excluded the small number of night-shift workers. Although our sample was representative for offshore day-shift workers, e.g. regarding age and high levels of good perceived health [41], the generalizability to night and swing shift workers is limited. A previous study by Merkus et al. (2017) showed that night and swing shift workers were more physically active than day workers during the leave period at home [41]. Thus, although most offshore workers work day-shifts rather than night-shifts, future studies could also be conducted among night shift workers, or among swing shift workers. Furthermore, during the offshore period, no differentiation between on and off-shift activity was made, i.e. activities during and beyond work hours. Participants worked 12-h shifts from 7 AM to 7 PM and our actigraph device measured day time activity between 6 AM and 11 PM. Thus, on- (7 AM–7 PM) and off-shift (6 AM–7 AM; 7 PM–11 PM) activity values offshore were incorporated into one activity measure. Furthermore, in our study, the same actigraph measures were used to measure objective PA at home and at work, while the type of PA differed. Future research could additionally examine specific types of activity, e.g. sports or lifting heavy loadings at work, to be able to examine which type of activity contributes to the development of fatigue. Lastly, as we used a continuous score to measure PA without intensity indication, we cannot conclude whether workers’ activity levels were below or above the recommended guidelines of being moderately physically active for at least 150 min or vigorously active for at least 75 min per week.

### Implications

Our study findings indicate that PA-levels are similar during the offshore work period and leave periods at home. In addition, results indicate that promoting physical activity among offshore workers will likely not contribute to reducing sleepiness in the offshore environment. This means that PA can be promoted for its health benefits but that sleepiness within this context needs further research. As PA is expected to be low within the offshore environment, increasing PA could prevent or reduce overweight and potentially reduce the prevalence of OSA. Future studies should focus on differences between on- and off-shift activity during the offshore period to separate out possible differences between leisure and non-leisure (occupational) PA. This would give insight into whether physical activity should be promoted during work hours or after. Furthermore, future research has to incorporate measures on the intensity and duration of PA, to be able to classify workers as active or inactive according to common PA guidelines. This may enable the identification of workers with low PA-levels, which could be at risk for adverse health outcomes.

## Conclusion

The study showed that the courses of PA did not differ during the pre-, offshore and post-offshore periods, and that PA was thus similar during the offshore work period and the pre- and post-offshore periods at home. Highest PA levels were found at day 1 and 7, the first and last day of the pre-offshore period, and lowest PA levels were observed at day 20, the last day of the offshore period. Furthermore, PA was not associated with sleepiness during the offshore shift rotation period.

## Data Availability

The datasets generated and/or analysed during the current study are not publicly available. The original datasets are stored at the Nederlandse Aardolie Maatschappij B.V. and Royal Dutch Shell, Assen, The Netherlands.

## Abbreviations

BMI: Body Mass Index
DAA: Daytime Activity Average
KSS: Karolinska Sleepiness Scale
LMM: Linear Mixed Models
MW: MotionWatch
NAA: Night-time Activity Average
SF: Short-Form Survey
PA: Physical activity
